# Bias in the prediction of genetic gain due to mass and half-sib selection in random mating populations

**DOI:** 10.1590/S1415-47572009005000064

**Published:** 2009-09-01

**Authors:** José Marcelo Soriano Viana, Vinícius Ribeiro Faria, Admilson da Costa e Silva

**Affiliations:** Departamento de Biologia Geral, Universidade Federal de Viçosa, Viçosa, MGBrazil

**Keywords:** predicted genetic gain, realized genetic gain, recurrent selection

## Abstract

The prediction of gains from selection allows the comparison of breeding methods and selection strategies, although these estimates may be biased. The objective of this study was to investigate the extent of such bias in predicting genetic gain. For this, we simulated 10 cycles of a hypothetical breeding program that involved seven traits, three population classes, three experimental conditions and two breeding methods (mass and half-sib selection). Each combination of trait, population, heritability, method and cycle was repeated 10 times. The predicted gains were biased, even when the genetic parameters were estimated without error. Gain from selection in both genders is twice the gain from selection in a single gender only in the absence of dominance. The use of genotypic variance or broad sense heritability in the predictions represented an additional source of bias. Predictions based on additive variance and narrow sense heritability were equivalent, as were predictions based on genotypic variance and broad sense heritability. The predictions based on mass and family selection were suitable for comparing selection strategies, whereas those based on selection within progenies showed the largest bias and lower association with the realized gain.

## Introduction

More than two decades ago, Wricke and Weber (1986) stated that the formula for predicting gain from selection “is certainly one of the central points in plant breeding research”. However, it is unlikely that either of these authors would now defend this position. Various relevant methods, such as selection indices, diallel analysis, stability and adaptability analysis, Best Linear Unbiased Prediction (BLUP) and QTL analysis, were developed by quantitative geneticists prior and after the proposition of a general function for gain prediction by Eberhart (1970). The prediction function developed by Eberhart (1970) based on work by Falconer (1960) has proven useful for assessing the efficiency of breeding methods and selection strategies. Although regularly used in breeding studies, this function, popularly known as ‘the breeder's equation', is not the only one available to quantitative geneticists (Loywyck *et al.*, 2005).

Gonçalves *et al.* (2007) assessed several selection processes in families of yellow passion fruit obtained by Design I. The best process was combined selection. The predicted gain from combined selection based on the number of fruits per plant was 18.55%, whereas the best results for index-based selection were 15.92% for Pesek and Baker and 15.85% for Mulamba and Mock. In a study with BR 5011 corn cultivar in which three mass selection cycles and 17 cycles of half-sib selection were used, Carvalho and Souza (2007) predicted an average gain in yield of 2.56% in the last 14 cycles. Rose *et al.* (2007) assessed the efficiency of half-sib selection in switchgrass (*Panicum virgatum* L.) in high and low yield environments. The predicted gains for dry matter were generally lower in the unfavorable environment. The predicted gain for family selection was superior to that for mass selection. Baltunis *et al.* (2007) showed that in loblolly pine (*Pinus taeda* L.) the predicted direct gain from half-sib selection for rooting ability was 36% while the predicted indirect gain for height at two years was 5.4%. Selecting the best families for height resulted in direct and indirect predicted gains of 8.1% and 14.8%, respectively. The predicted direct gain from full-sib selection for rooting ability was 43% while the predicted indirect gain for height at two years was 9%. Selecting the best families for height yielded direct and indirect predicted gains of 10.1% and 8.6%, respectively. The selection of clones based on rooting ability resulted in a predicted direct gain of 96% associated with a decrease in height. Selecting the best clones for height resulted in direct and indirect predicted gains of 27% and 43%, respectively. Thus, overall, the selection indices assessed resulted in gains for both traits.

Despite its usefulness in helping to choose the best breeding method or selection process, the Eberhart prediction formula is widely known to provide a biased estimate (usually an overestimate) of changes in the population mean. Bordes *et al.* (2006) compared the efficiency of two methods of corn inbred lines development. For yield, the use of the dihaploid method resulted in a predicted gain of 2%/year, which was lower than the predicted gains of 2.4%/year and 2.9%/year for two cycles of S_1_ families, respectively, in four years. The real gains were 1.65%/year and 1.75%, respectively, indicating overestimation of the predicted gains. A study with popcorn showed that although there was agreement between the predicted and true mean gains in expansion volume and yield, the predictions per cycle were generally overestimated (Viana, 2007). Similar results are reported by Hallauer and Miranda Filho (1988).

In view of the lack of information on the relative importance of possible sources of bias, the aim of this study was to investigate biases in the prediction of genetic gains from selection.

## Material and Methods

###  Sources of bias in the prediction of genetic gains

Although generally applicable to genetic breeding, the Eberhart function is based on mass selection in a single gender. The genetic gain (Δ*M*) is calculated as *M*_1_ - *M*, where *M*_1_ is the genotypic mean of the bred population and *M* is the genotypic mean of the population in which the selection was made. The gain is proportional to the difference between the phenotypic mean of the selected population $\left({\bar{P}}_{s}\right)$ and the phenotypic mean of the base population $\left(\bar{P}\right)$, referred to as the selection differential (*SD*), *i.e.*, $\Delta M=b\cdot ({\bar{P}}_{s}-\bar{P})$.  Thus, *M*_1_ = *M* + *b*.*SD*. Since the bred population consists of half-sib families whose common parents are the selected individuals, the parameter b should be the same as the regression of the mean phenotypic value of progeny as a function of the difference between the phenotypic value of the selected individual and the phenotypic mean of the base population ( 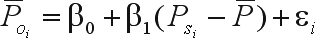, assuming identity of the models for each selected individual). Based on this assumption, 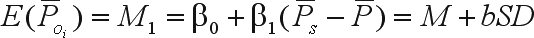. In addition, assuming that genotypic value and environmental effect are independent,



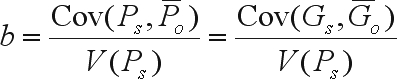


where *G*_*s*_ and ${\bar{G}}_{o}$are the genotypic values of a selected individual and its progeny in the bred population, the covariance of which is unknown. Assuming that alterations in the gene frequencies are negligible, then in the case of the additive-dominant model (Wricke and Weber, 1986)



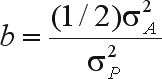


where 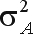 and 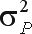 are the additive genetic variance and the phenotypic variance in the base population, respectively.

Hence, the predicted gain from mass selection on a single gender is



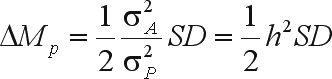


where *h*^2^ is the heritability.

Introducing selection intensity (*i* = *SD*/σ_*P*_) (Wricke and Weber, 1986) results in



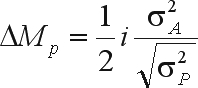


where 1/2 is the parental control (Eberhart, 1970).

Assuming that the numerator of the coefficient of proportionality b is the covariance between the additive genetic value of an individual in the selection unit (X) and the additive genetic value of its relative in the bred population (Y) (COV_A_(X, Y) = 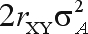, where *r*_XY_ is the coefficient of relationship between X and Y), then the predicted gain in a year is (Eberhart, 1970)



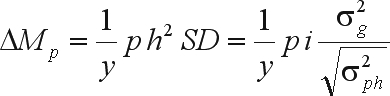


where *p* is the parental control (1/2, 1 or 2), *h*^2^ is the heritability of the selection unit, 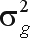 is the genotypic variance of the selection units, attributable to the average effects of the genes ( 
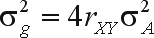
), 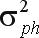 is the phenotypic variance of the selection units and y is the number of years per cycle. This is a generalization of the function presented by Falconer (1960) for mass selection on both genders.

Since the additive covariance between an individual in the selection unit and its relative in the bred population is only equal to 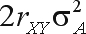 in the case of absence of selection, the genetic gain prediction function is biased because even though the selection is not efficient the prediction will not necessarily be nil. Additional biases will result from errors in estimating *h*^2^ and 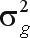, attributable to sampling, experimental error and unmet assumptions such as Hardy-Weinberg equilibrium, linkage equilibrium and the absence of epistasis.

###  Theoretical genetic gains

For a single gene and mass selection on only one gender in a population under Hardy-Weinberg equilibrium, the probabilities of the genotypes in the group of selected individuals are



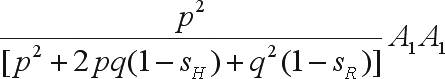




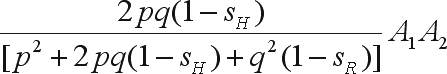




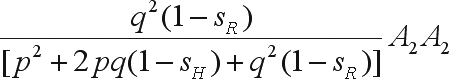


where *p* and *q* are the frequencies of the A_1_ and A_2_ alleles in the base population, *s*_*H*_ is the intensity of selection against the heterozygote (carrier of the undesirable A_2_ gene) and *s*_*R*_ is the selection intensity against the homozygote for the unfavorable allele. The function



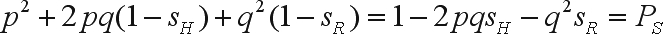


is the proportion of selected individuals, which is a function of the initial gene frequencies and of the *s*_*H*_ and *s*_*R*_ values. With no selection (natural or artificial), *s*_*H*_ = *s*_*R*_ = 0 and, therefore, *P*_*S*_ = 1.

The change in the frequency of the favorable gene is



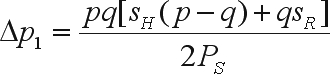


The mean of the improved population is



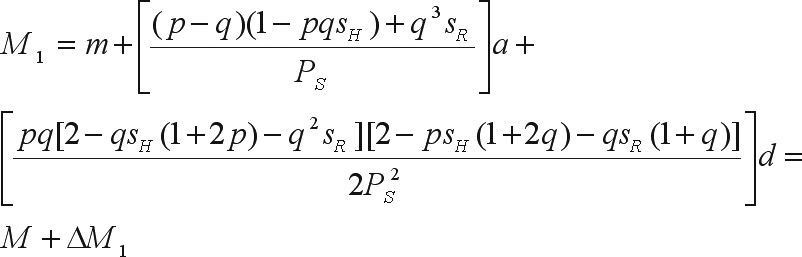


where *m* is the mean of the genotypic values of the homozygotes, *a* is the deviation between the genotypic value of the homozygote of greater expression and *m*, *d* is the deviation between the genotypic value of the heterozygote and *m*, and *M* = *m* + (*p* - *q*)*a* + 2*pqd* is the mean of the base population (Wricke and Weber, 1986).

The genetic gain due to selection is



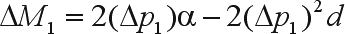


where α is the effect of substituting the A_2_ gene with the A_1_ gene (Wricke and Weber, 1986). Since the selection intensity is the ratio between the height of the ordinate of the standard normal distribution corresponding to the truncating point (*a*_*t*_) and the proportion of selected individuals (*P*_*S*_) (Wricke and Weber, 1986), then



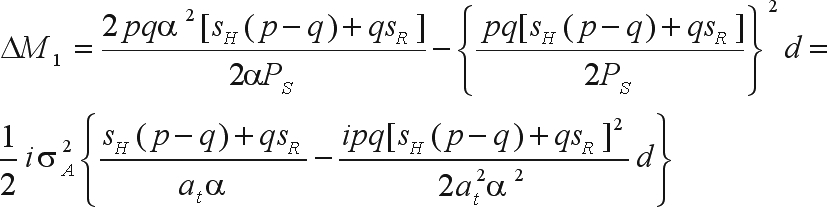


The bias in the genetic gain prediction is



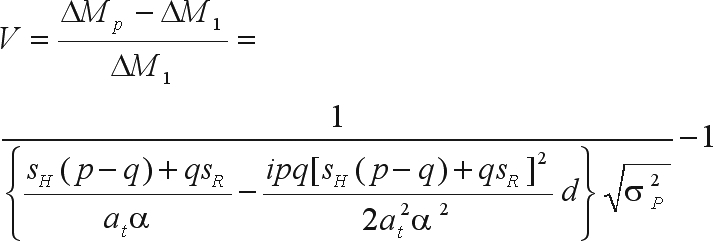


With mass selection on both genders the change in the frequency of the favorable gene is Δ*p*_2_ = 2Δ*p*_1_. The mean of the bred population is



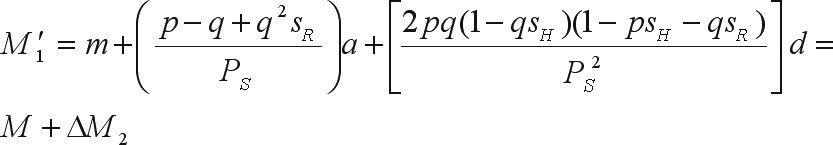


Thus,



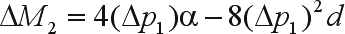


or,



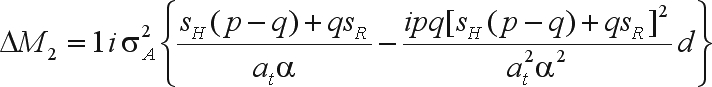


The bias in the prediction of genetic gain is



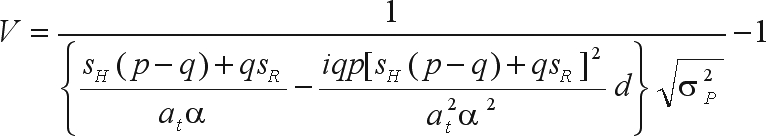


The Δ*M*_2_/Δ*M*_1_ ratio is only equal to two if there is no dominance and, accordingly, only in this situation will the gain from mass selection on the two genders be twice the gain from mass selection on a single gender. Therefore, if dominance is present, then the assumption that selection on both genders results in a predicted gain that is two-fold greater than for selection on only one of the genders is an approximation and a further source of bias.

The impossibility of using the bias functions to investigate the magnitude of bias must be emphasized since the selection intensities for *s*_*H*_ and *s*_*R*_ on each gene, together with the selection intensity i, are not known *a priori*. The same is true for family selection. In the case of half-sib selection with recombination only among individuals of the selected progenies, the alteration in the favorable gene frequency is



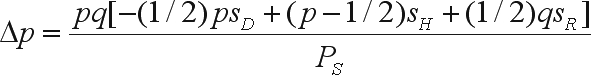


where *s*_*D*_, *s*_*H*_ and *s*_*R*_ are the selection intensities on the families of common parents A_1_A_1_, A_1_A_2_ and A_2_A_2_.

The mean of the bred population, based on a recombination generation after the selection cycle, is



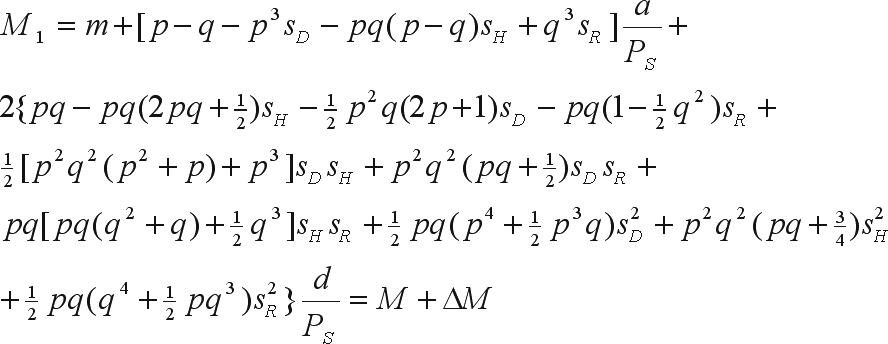


The genetic gain from among family selection is



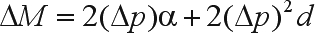


or,



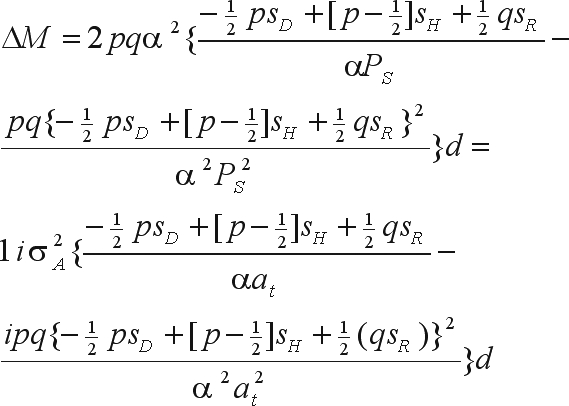


The bias in the genetic gain prediction is



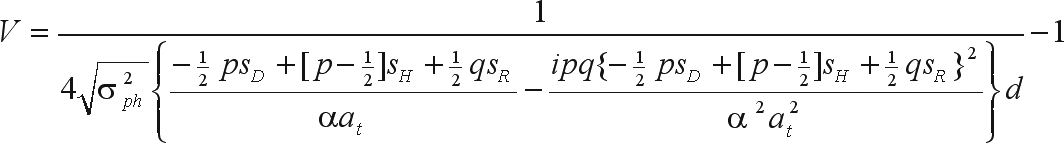


###  Characterization of the gene systems, populations and environmental conditions

The simulation done here considered seven generic traits, three classes of populations, three environmental conditions and two breeding methods, both conducted for 10 cycles. The traits were characterized by different degrees of dominance. The values 2 and -2, 1 and -1, 0.5 and -0.5, and 0 were used to define overdominance, complete dominance, partial dominance and no dominance, respectively. A positive value indicated dominance of a favorable gene (one that increased trait expression) whereas a negative value indicated dominance of the unfavorable gene (one that decreased trait expression). Each trait was assumed to be determined by 10 genes with an assortative distribution. Additional assumptions included absence of epistasis, Hardy-Weinberg equilibrium and linkage equilibrium.

Since the frequencies of favorable genes in a population can range from 0 to 1, we attempted to represent all possible populations by using three categories, namely, an unimproved population, a population with intermediate frequencies of favorable genes and an improved population. The frequencies of the favorable genes for these classes were assumed to be 0.1, 0.5 and 0.9, respectively. The experimental conditions or degree of error control also varied, which resulted in changes in the parametric values for heritability based on the magnitude of the environmental effects that were introduced. This approach accounted for situations of high (90%), intermediate (50%) and low (10%) heritability. Because of the difficulty in precisely establishing the desired heritability value, a variation of ±4% in the desired value was allowed. The breeding methods used were mass selection in one sex and half-sib selection with recombination of selected progenies. In the case of mass selection, the population size was 1000. With half-sib selection, the simulation assumed 200 progenies (200 females and an infinite male gamete pool), with a completely randomized block design, two replications and 25 individuals per plot. The best 10% were selected based on phenotypic values of the individuals and the average phenotypic values of the families. For the recombination plot, the simulation assumed 100 individuals in each selected progeny and an infinite male gamete pool.

The genetic gain due to mass selection was calculated as the difference between the parametric mean of the improved population (cycle n + 1) and the mean of the previous population (cycle n). The genetic gain due to family selection was calculated as the difference between the mean of the improved population obtained with family selection and the mean of the previous population, whereas the gain for the selection of superior individuals in the best progenies was calculated as the difference between the mean of the improved population obtained by among and within selection and the mean of the improved population obtained with family selection. A generation of random mating was assumed to occur after each selection cycle. The predicted gains were calculated based on the parametric values of additive and genotypic variances and of narrow and broad sense heritabilities, as well as the estimated values of these parameters. With mass selection, there was no defined constant bias in the estimate of the additive variance. The estimates were also obtained by simulating parent-offspring and mid-parent-offspring regressions (average of 10 estimates for each regression). In the case of half-sib selection, the estimates of additive variance came from analyses of variance. The function of the predicted gain due to within family selection was



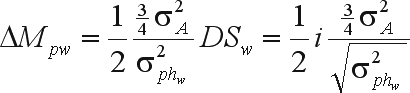


or



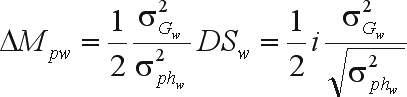


The simulated data were obtained by using many of the built-in functions of Microsoft Excel^®^ software (Microsoft Inc.). The sequence of events used was: (1) specification of the trait and effects of the favorable genes, with insertion of the degree of dominance (the same for each gene), (2) characterization of the population, with insertion of the frequencies of the favorable genes, (3) specification of the environmental conditions, with definition of the desired heritability, (4) calculation of the population parametric mean (cycle 0), (5) simulation of the individual genotypes in the case of mass selection, or of the parent genotypes (females) and 150 individual genotypes for each progeny in the case of half-sib selection, (6) simulation of the genotypic values, environmental effects and phenotypic values of the individuals, (7) in the case of half-sib selection, analysis of variance of the plot phenotypic values (mean phenotypic value of 25 individuals), (8) estimation of genetic parameters (genotypic and additive variances, and heritabilities) and prediction of gains, (9) identification of superior individuals in the case of mass selection, or of the best families in the case of half-sib selection, (10) computation of the gene frequencies in the improved population, and (11) computation of the improved population mean and of realized gains (first cycle). For the other cycles, the same order of events was used, except for events (1) and (3). Note the correspondence between events (10) and (11) for cycle n and events (2) and (4) for cycle n + 1.

Each combination of trait (7), population (3), heritability (3), breeding method (2) and cycles (10) was repeated ten times and corresponded to 12.600 simulations. In the case of mass selection, when the predicted gain was calculated with estimates of the parameters (biased estimates) only one replication was done (total of 1.260 simulations).

## Results and Discussion

###  Mass selection

Few experimental studies have compared predicted and realized gains, especially using mass selection. This lack of data makes it difficult to compare the results for biases in gain predictions with mass selection (Table 1). Another limiting factor, even when experimental data are available, is the lack of knowledge about gene frequencies in the population under selection, *i.e.*, the level of breeding in the population and the degree of dominance of the genes controlling the traits being studied. As shown here, the prediction of gain from selection is biased, even when the true values of the genetic parameters (unbiased estimates) are used in the calculation. Ignoring biases > 300% that essentially reflected only a small predicted gain and no actual gain, the mean biases in this simulation ranged from 39.2% to 59.3%, depending on the prediction function used. Extreme values generally represented < 10% of the cases and occurred mainly in bred populations with average heritability.

Overestimation of gain was not a general rule in our analysis. When additive variance or narrow sense heritability was used there was a tendency to underestimate the gain, particularly with low heritability (Table 1). However, when genotypic variance or broad sense heritability was used, the overestimation of gain for traits with a mean dominance =1.0 was more frequent. Consequently, the use of genotypic variance or broad sense heritability (rather than additive variance and narrow sense heritability) was a further source of bias in gain prediction. In several cases, the bias went from negative (underestimation) to positive (overestimation) values, with an increase in magnitude. The mean absolute values of the biases ranged from 39.2% to 59.3% (increase of 51.3%) with the use of genotypic variance, and from 41.3% to 49.9% (increase of 20.8%) with the use of broad sense heritability. The magnitude and sign of the biases further showed that prediction based on selection intensity and additive variance was equivalent to prediction based on narrow sense heritability and selection differential (means absolute values of the biases were 39.2% and 41.3%, respectively). The same was true for the use of genotypic variance and broad sense heritability (means of 59.3% and 49.9%, respectively).

The results of different traits showed that the magnitude of the bias was proportional to the degree of dominance, regardless of whether the favorable genes were dominant or recessive (Table 1). With prediction based on additive variance, the mean magnitude of the bias with complete dominance/overdominance and partial dominance/absence of dominance was 47.7% and 27.5%, respectively. Finally, small magnitude bias was observed in populations with intermediate frequencies and under high heritability conditions. The means of the absolute values were 42.2%, 42.9% and 32.9% for cases of low, medium and high heritability, respectively, and 51.9%, 27.5% and 37.9% in non-bred populations, populations with intermediate frequencies, and bred populations, respectively, with prediction based on additive variance.

Although the Eberhart function yielded biased estimates of genetic gain, our simulation indicated that this function was adequate for assessing the efficiency of recurrent population breeding methods and selection strategies. The correlation between realized and predicted genetic gains during 10 cycles was generally positive and of high magnitude (average of 0.84) (Table 2). The exceptions (values < 0.70), which represented 6%-12% of the cases analyzed, did not show any tendency and can be attributed to chance. Again, there was full correspondence between the results obtained with prediction using additive variance or narrow sense heritability and those obtained based on genotypic variance or broad sense heritability.

When gain is predicted based on biased estimates of the genetic parameters, the additional bias can increase or decrease the difference between the realized and predicted gains. Using estimates of additive variance (obtained by parent/offspring and mid-parent/offspring regressions) and genotypic variance (obtained from the difference between the phenotypic and environmental variances), the simulation study confirmed almost all of the previous inferences. The exception was a small bias in a bred population, for which the mean magnitude ranged from 27.4% to 47.9%, depending on the prediction function. The bias in the estimates of additive variance ranged from -30.1% to 24.6%, with a predominance of underestimation (71.4% of the cases), which explained the smaller magnitude of the bias observed here. With few exceptions, the realized and predicted gains during 10 cycles were also in full agreement (average correlation of 0.80).

###  Half-sib selection

The results of bias in predictions of gain from family selection showed similarities and differences compared to those obtained with mass selection (Table 3). Although the amplitude of the absolute value of bias was not smaller (minimum of 0.25% and maximum of 158.4%, with prediction based on the parametric value of the additive variance), there were no very high results (> 300%) and the mean value was 17.7%. The corresponding values in the case of mass selection were 0.31%, 149.1% and 39.2% (Table 1). Although the frequency of cases involving overestimation and underestimation were equivalent (54% and 46%), there was a tendency for overestimation in traits controlled by dominant favorable genes. These results were similar to the findings of Carvalho *et al.* (2000) for corn yield, in which the bias between the predicted and realized gains was 287.3%. Bonomo *et al.* (2000) reported yield biases of 53.5%, 119.0%, 129.8% and 88.3% when the selection intensity varied from the lowest to the highest value. More recently, Viana (2007) calculated the realized gain by using the means of the progeny tests and observed full correspondence between the realized and predicted gains. The respective means of the predicted and realized gains for the three selection cycles were 5.6% and 5.6% for expansion volume, and 8.1% and 7.8% for yield.

The mean absolute values of biases by trait, population and heritability were larger with complete dominance and overdominance (21.4%), in bred populations (28.9%) and with low heritability (23.8%) (Table 3). The mean values in cases of partial/absence of dominance, in non-bred populations and in populations with intermediate gene frequencies, average heritability and high heritability were, respectively, 12.7%, 12.3%, 11.9%, 14.7% and 14.6%. Once again, equivalence was observed between prediction based on selection intensity and additive variance and prediction based on narrow sense heritability and selection differential. For bias in the predictions based on estimates of additive variance, all of the previous inferences were confirmed, with no exceptions (Table 3). Although the use of biased estimates of genetic parameters can either increase or decrease the bias calculated based on parametric values, only increases were observed here. The absolute minimum, mean and maximum values were 0.52%, 25.9% and 180.4%, respectively.

The gain prediction from family selection was a poorer indicator of the efficiency of recurrent population breeding methods and selection strategies compared to similar prediction from mass selection (Table 4). The linear association between predicted and realized gains during 10 cycles was only adequate for heritability > 50%, regardless of the traits and the bias in the additive variance estimates. The mean correlation was 0.71 for prediction based on unbiased estimates of the additive variance, and 0.59 in the case of prediction based on biased estimates. When low heritability cases were excluded, the mean correlations were 0.85 and 0.81.

Comparison of the predicted and realized gains based on the selection of individuals in the best families yielded poor results (Tables 3  and 4). In approximately 54% of the cases, the realized gain was practically nil, implying very high bias values in relation to the predicted gain (Table 3). This situation occurred in predictions of traits controlled by dominant favorable genes (degree of dominance > 0, regardless of the bias in the estimates of additive variance). When these values were ignored, the smallest magnitude of bias was 4.6% and the absolute maximum value was 297.9%, with prediction using unbiased estimates of additive variance. The mean magnitude of the bias was 94.5%. These values were greater than those observed with mass selection, indicating that prediction of gain from within half-sib selection is more biased than prediction of gain from mass selection using unbiased estimates of additive variance. There was a tendency for underestimation in traits controlled by favorable genes with a degree of dominance > 1.0, as also seen with corn grain yield. However, overestimation was detected in the other situations. These observations agreed with findings for popcorn yield (Matta and Viana, 2003), for which the biases in gain predictions from among and within selection were 218.1% and -116.3%, respectively, in line with the theoretical results. For expansion volume, considered by Scapim *et al.* (2002) to be determined by favorable dominant and recessive genes (bi-directional dominance), the bias was towards underestimation, *i.e.*, -18.4% with progeny selection and -78.4% with selection of individuals in the selected families. As expected, bias in gain prediction from within selection was much larger than bias in gain prediction from among family selection.

Greater biases were observed for traits controlled by favorable dominant genes (average magnitudes of 133.3%, 297.9% and 115.0% for degrees of dominance of 0.5, 1 and 2, respectively) and traits not controlled by allelic interaction effects (average magnitude of 143.8%) (Table 3). The average absolute values of the biases for traits controlled by favorable recessive genes were 90.1%, 54.9% and 41.0% for degrees of dominance of -0.5, -1 and -2, respectively. Greater absolute biases were observed in populations with intermediate gene frequencies (113.1% versus 81.6% and 86.2%, in non-bred and bred populations) and low heritability (108.8% versus 82.4% and 92.4%, with medium and high heritability). The predicted gains calculated based on biased estimates of additive variance were more biased, but generally confirmed the results obtained by using the parametric value. The minimum, mean and maximum magnitudes were 4.1%, 102.3% and 296.1%, respectively.

An additional negative aspect of gain prediction from individual selection within the selected families was shown by the correlation between predicted and realized gain during 10 cycles. Regardless of the magnitude of the bias in additive variance, the correlation was negative in ~40% of the situations assessed (Table 4) but was > 0.7 in only 30%-40% of the cases. Only in cases of traits controlled by favorable recessive genes with average to high heritability was there sufficient agreement between predicted and realized gains to allow assessment of the efficiency of the recurrent breeding method and selection strategies (average correlation of 0.75, regardless of the bias in the additive variance estimate). The average correlations for unbiased and biased estimates of the additive variance were 0.15 and 0.24, respectively.

In conclusion, the use of unbiased and biased estimates of the genotypic variance within progeny rather than the within family additive variance, *i.e.*, broad versus narrow sense heritability, increased the magnitude of bias without worsening the correlation between predicted and realized gains. These findings indicate that Eberharts formula, which is a function of additive variance or narrow sense heritability, is a less biased estimator of genetic gain than the estimator based on a function of genotypic variance or broad sense heritability. As shown for mass and family selection, there was full correspondence between the gains calculated with additive or genotypic variance and the predictions based on broad or narrow sense heritability.

## Figures and Tables

**Table 1:** - Percentage bias between realized and predicted gains in the first mass selection cycle based on unbiased estimates of additive and genotypic variances^1^.

Gain	*h*^2^ (%)	p	Degree of dominance						
			2	1	0.5	0	-0.5	-1	-2
		0.1	-41.19	-42.63	-40.52	-44.06	-39.11	-44.15	-53.18
	10	0.5	-15.2	-28.65	-30.73	-36.28	-45.97	-47.91	-60.90
		0.9	-127.6	0.31	-19.62	-25.00	-24.36	-64.11	-53.99
	
		0.1	-13.37	-10.76	-80.60	-55.24	-23.70	-57.83	129.21
Δ*M*_*p*__1_	50	0.5	32.71	11.67	5.16	-1.28	-7.78	-34.41	-85.93
		0.9	-24.31	-114.2	-776.4	-360.9	-843.3	40.99	1445.47
	
		0.1	38.86	36.45	-51.86	23.11	20.22	-95.20	-149.1
	90	0.5	49.99	26.03	10.52	2.13	-5.29	-11.38	-26.75
		0.9	-16.63	529.21	27.02	22.31	18.77	11.93	14.84

		0.1	-34.93	-39.44	-39.15	-44.06	-31.50	207.18	40.46
	10	0.5	154.39	7.02	-22.07	-36.28	-39.22	-21.88	17.29
		0.9	-182.9	451.70	-9.57	-25.00	-22.62	-62.11	-49.09
	
		0.1	-4.14	-5.80	-80.15	-55.24	-14.16	131.93	587.55
Δ*M*_*p*__2_	50	0.5	298.14	67.50	18.30	-1.28	3.75	-1.61	-57.78
		0.9	127.09	-178.2	-861.0	-360.9	-994.2	48.28	1610.1
	
		0.1	53.65	44.03	-50.76	23.11	35.25	-73.60	-247.5
	90	0.5	349.98	89.04	24.34	2.13	6.55	32.93	119.76
		0.9	150.10	3360.7	42.90	22.31	21.50	18.15	27.08

		0.1	-40.84	-42.82	-40.85	-46.03	-42.94	-69.64	-56.36
	10	0.5	-17.00	-31.56	-32.88	-39.14	-47.09	-48.18	-58.84
		0.9	-127.6	-8.92	-27.51	-29.21	-27.90	-63.97	-54.68
	
		0.1	-4.90	-5.69	-76.21	-51.06	-16.23	-50.33	130.41
Δ*M*_*p*__3_	50	0.5	33.54	11.14	4.49	-1.29	-4.74	-29.65	-83.57
		0.9	-23.56	-110.7	-670.9	-350.4	-966.4	30.55	1448.63
	
		0.1	52.74	49.61	-42.25	37.61	45.58	-86.19	-192.7
	90	0.5	45.06	19.34	4.76	1.04	-0.42	-6.19	-21.93
		0.9	-16.24	174.76	-2.07	5.67	5.42	1.01	3.47

		0.1	-34.53	-39.63	-39.51	-46.03	-35.80	190.91	30.93
	10	0.5	149.00	2.66	-24.49	-39.14	-40.48	-22.28	23.49
		0.9	-182.6	400.95	-17.33	-29.21	-26.25	-61.97	-49.85
	
		0.1	5.22	-0.46	-75.67	-51.06	-5.75	173.19	591.24
Δ*M*_*p*__4_	50	0.5	300.62	66.71	17.55	-1.29	7.17	5.53	-50.73
		0.9	129.32	-158.7	-742.3	-350.4	-976.9	37.25	1613.6
	
		0.1	69.01	57.93	-40.93	37.61	63.78	-24.07	-378.3
	90	0.5	335.17	79.01	17.86	1.04	12.03	40.71	134.21
		0.9	151.27	1411.2	10.17	5.67	7.84	6.62	14.49

^1^Δ*M*_*p*__1_, Δ*M*_*p*__2_, Δ*M*_*p*__3_, and Δ*M*_*p*__4_ are the predicted gains based on additive variance, genotypic variance, narrow sense heritability and broad sense heritability; *h*^2^ is the broad sense heritability and p is the frequency of the favorable gene.

**Table 2:** - Correlation between realized and predicted gains in 10 mass selection cycles based on unbiased estimates of additive and genotypic variances^1^.

Gain	*h*^2^ (%)	p	Degree of dominance						
			2	1	0.5	0	-0.5	-1	-2
		0.1	0.98	0.85	0.74	0.76	0.98	0.98	0.95
	10	0.5	0.75	0.99	0.90	0.96	0.96	0.54	0.68
		0.9	0.98	0.80	0.99	0.99	0.97	0.97	0.96
	
		0.1	0.99	0.99	0.96	0.88	0.89	0.98	0.95
Δ*M*_*p*__1_	50	0.5	0.98	1.00	0.99	0.99	0.98	0.98	0.98
		0.9	0.87	-0.26	1.00	1.00	1.00	1.00	1.00
	
		0.1	1.00	0.99	0.99	0.97	0.96	0.98	-0.07
	90	0.5	0.98	1.00	0.99	1.00	0.99	0.98	0.96
		0.9	0.79	0.18	0.99	0.98	0.97	0.98	0.97

		0.1	0.93	0.81	0.72	0.76	0.98	0.99	0.82
	10	0.5	0.75	0.98	0.90	0.96	0.95	0.53	-0.02
		0.9	-0.98	0.75	0.99	0.99	0.97	0.97	0.97
	
		0.1	0.97	0.97	0.96	0.88	0.94	0.98	0.82
Δ*M*_*p*__2_	50	0.5	0.97	0.99	0.99	0.99	0.98	0.98	0.80
		0.9	-0.92	-0.27	1.00	1.00	1.00	1.00	1.00
	
		0.1	0.98	0.96	0.98	0.97	0.97	0.98	0.22
	90	0.5	0.97	0.98	0.98	1.00	1.00	0.99	0.88
		0.9	-0.91	0.13	1.00	0.98	0.97	0.99	0.98

		0.1	0.98	0.84	0.76	0.83	0.97	0.99	0.94
	10	0.5	0.73	0.99	0.92	0.96	0.96	0.60	0.70
		0.9	0.98	0.80	0.99	0.98	0.97	0.97	0.97
	
		0.1	0.99	0.99	0.98	0.92	0.94	0.99	0.94
Δ*M*_*p*__3_	50	0.5	0.98	1.00	0.99	0.99	0.99	0.99	0.99
		0.9	0.88	-0.33	1.00	1.00	0.99	1.00	1.00
	
		0.1	1.00	1.00	1.00	0.99	0.99	0.99	-0.06
	90	0.5	0.98	1.00	0.99	1.00	1.00	0.99	0.98
		0.9	0.81	0.02	0.99	0.98	0.99	0.99	0.98

		0.1	0.94	0.79	0.74	0.83	0.97	0.98	0.80
	10	0.5	0.71	0.98	0.92	0.96	0.96	0.56	0.01
		0.9	-0.98	0.75	0.99	0.98	0.97	0.97	0.97
	
		0.1	0.97	0.97	0.98	0.92	0.98	0.95	0.81
Δ*M*_*p*__4_	50	0.5	0.96	0.99	0.99	0.99	0.99	0.97	0.79
		0.9	-0.92	-0.38	1.00	1.00	0.99	1.00	1.00
	
		0.1	0.99	0.99	0.99	0.99	0.95	0.92	0.25
	90	0.5	0.95	0.99	0.99	1.00	1.00	0.99	0.85
		0.9	-0.87	-0.12	0.99	0.98	0.99	0.99	0.99

^1^Δ*M*_*p*__1_, Δ*M*_*p*__2_, Δ*M*_*p*__3_, and Δ*M*_*p*__4_ are the predicted gains based on additive variance, genotypic variance, narrow sense heritability and broad sense heritability; *h*^2^ is the broad sense heritability and p is the frequency of the favorable gene.

**Table 3:** - Percentage bias between realized and predicted gains in the first half-sib selection cycle based on unbiased and biased estimates of additive variance^1^.

Gain	*h*^2^ (%)	p	Degree of dominance						
			2	1	0.5	0	-0.5	-1	-2
		0.1	-19.00	-20.47	-6.16	4.38	-27.90	-27.39	-10.96
	10	0.5	37.30	0.82	41.41	4.88	-9.23	-16.68	-4.44
		0.9	31.28	56.77	-5.15	10.52	3.35	158.35	2.66
	
		0.1	-5.96	7.50	-15.53	-2.18	-22.79	-25.70	5.39
Δ*M*_*p*__1_	50	0.5	21.83	18.82	-1.14	2.81	-1.28	-1.63	-7.81
		0.9	22.03	38.40	16.35	21.01	29.40	25.80	16.37
	
		0.1	-0.25	-4.25	-3.75	-11.81	-12.80	-22.63	2.64
	90	0.5	33.46	12.98	21.03	-0.31	-1.35	-1.14	-9.30
		0.9	31.67	38.15	24.63	24.80	17.40	12.45	19.66

		0.1	18.39	-9.94	-19.04	47.30	-48.20	-21.55	36.01
	10	0.5	25.14	180.37	61.64	4.31	-42.59	3.32	-17.67
		0.9	-45.16	121.57	12.89	-19.22	43.73	300.75	72.21
	
		0.1	-15.15	-18.67	-9.95	-11.02	-18.57	-29.11	20.56
Δ*M*_*p*__2_	50	0.5	19.15	10.10	5.34	-1.98	6.07	-10.42	-17.38
		0.9	16.39	48.87	30.79	16.99	26.74	24.12	26.70
	
		0.1	-4.61	-7.65	35.16	-7.55	-12.96	-30.79	1.62
	90	0.5	28.11	-18.57	38.62	0.52	-4.74	-2.13	-16.23
		0.9	31.05	35.77	24.77	26.48	15.03	16.05	11.49

		0.1	-2022.8	1765.9	2859.3	-2731.6	207.61	24.61	54.26
	10	0.5	-174.85	-360.78	-997.85	241.01	148.74	112.28	66.30
		0.9	-46.94	-297.91	66.34	62.33	44.97	42.59	40.95
	
		0.1	319.24	2179.0	734.11	247.40	50.33	-40.30	26.60
Δ*M*_*p*__3_	50	0.5	-210.31	-542.20	470.02	140.62	81.73	43.97	4.06
		0.9	-22.44	-1122.4	98.64	87.94	66.67	59.97	54.67
	
		0.1	2868.4	-10233	16318	141.17	47.42	-49.49	8.95
	90	0.5	-223.79	-1221.0	-156.49	116.02	47.63	16.22	-25.64
		0.9	-11.61	-18235	211.86	114.09	115.74	104.66	87.21

		0.1	-2716.3	292.01	1996.1	-5148.0	148.85	32.52	135.68
	10	0.5	-169.52	-438.32	-2715.7	217.67	44.14	184.88	50.12
		0.9	-74.78	-296.15	56.51	71.19	79.57	78.37	160.09
	
		0.1	301.62	1635.2	682.26	231.01	61.11	-41.63	46.30
Δ*M*_*p*__4_	50	0.5	-211.79	-510.10	485.40	133.55	101.25	34.65	-4.10
		0.9	-24.73	-1332.6	98.66	83.42	66.33	60.45	68.45
	
		0.1	2288.1	-8804.4	18403	155.97	47.06	-53.35	8.99
	90	0.5	-219.65	-1234.3	567.79	119.05	43.91	15.70	-30.96
		0.9	-10.33	-17028	217.45	120.26	112.30	113.96	75.96

^1^Δ*M*_*p*__1_ and Δ*M*_*p*__2_ are the predicted gains with family selection, calculated based on the parametric and estimated values of additive variance; Δ*M*_*p*__3_ and Δ*M*_*p*__4_ are the predicted gains with selection of individuals in the best progenies, calculated based on the parametric and estimated values of additive variance; *h*^2^ is the narrow sense heritability and p is the frequency of the favorable genes.

**Table 4:** - Correlation between realized and predicted gains during 10 half-sib selection cycles based on unbiased and biased estimates of additive variance^1^.

Gain	*h*^2^ (%)	p	Degree of dominance						
			2	1	0.5	0	-0.5	-1	-2
		0.1	0.01	0.96	-0.21	-0.19	0.51	0.90	0.92
	10	0.5	0.90	1.00	0.43	-0.08	-0.09	-0.01	0.45
		0.9	-0.94	0.99	0.62	0.53	0.85	0.84	0.82
	
		0.1	0.95	0.88	0.63	0.55	0.85	0.92	0.99
Δ*M*_*p*__1_	50	0.5	0.96	1.00	0.83	0.97	0.95	0.98	0.94
		0.9	-0.30	1.00	1.00	1.00	1.00	0.99	0.99
	
		0.1	0.72	0.97	0.99	0.98	0.53	0.53	0.83
	90	0.5	0.96	0.99	0.99	0.99	0.86	0.69	0.96
		0.9	-0.45	0.99	0.98	0.96	1.00	1.00	1.00

		0.1	0.07	0.27	0.31	-0.06	0.67	0.88	0.57
	10	0.5	0.55	-0.29	0.10	0.24	0.13	-0.17	-0.44
		0.9	-0.01	-0.28	0.34	-0.06	0.02	-0.07	0.34
	
		0.1	0.95	1.00	0.75	0.52	0.93	0.93	0.94
Δ*M*_*p*__2_	50	0.5	0.73	0.32	0.60	0.95	0.75	0.97	0.94
		0.9	0.46	0.73	0.89	0.94	0.97	0.86	0.96
	
		0.1	1.00	1.00	0.99	0.99	0.99	0.99	0.95
	90	0.5	-0.17	0.96	0.99	0.99	1.00	0.99	0.96
		0.9	-0.20	0.98	0.99	0.96	0.49	0.76	0.37

		0.1	0.34	-0.98	0.05	-0.75	0.64	0.95	0.99
	10	0.5	-0.74	-0.97	-0.52	0.13	-0.65	-0.76	-0.50
		0.9	0.87	-1.00	-0.63	-0.24	0.01	0.46	0.57
	
		0.1	-0.98	-0.96	-0.04	-0.67	0.73	0.86	0.96
Δ*M*_*p*__3_	50	0.5	-0.88	-0.98	-0.96	-0.64	-0.53	0.57	0.86
		0.9	0.94	-0.89	0.93	0.97	0.99	0.99	1.00
	
		0.1	-0.83	0.28	0.62	0.96	0.70	0.56	0.71
	90	0.5	-0.94	-0.95	-0.63	0.95	0.94	0.61	0.62
		0.9	0.93	0.59	1.00	0.95	1.00	1.00	1.00

		0.1	0.35	-0.26	0.55	-0.29	0.67	0.88	0.46
	10	0.5	-0.58	0.33	-0.11	0.04	-0.21	-0.22	-0.06
		0.9	-0.13	0.31	-0.14	0.04	0.31	-0.11	-0.33
	
		0.1	-0.89	-0.98	0.11	-0.57	0.60	0.84	0.94
Δ*M*_*p*__4_	50	0.5	-0.73	-0.36	-0.87	-0.49	-0.48	0.52	0.89
		0.9	0.38	-0.84	0.89	0.93	0.97	0.87	0.96
	
		0.1	-0.63	0.31	0.68	0.96	0.97	0.97	0.79
	90	0.5	0.13	-0.93	-0.59	0.93	0.99	0.98	0.89
		0.9	0.32	0.58	0.99	0.94	0.53	0.82	0.39

^1^Δ*M*_*p*__1_ and Δ*M*_*p*__2_ are the predicted gains with family selection, calculated based on the parametric and estimated values of additive variance; Δ*M*_*p*__3_ and Δ*M*_*p*__4_ are predicted gains with selection of individuals in the best progenies, calculated based on the parametric and estimated values of additive variance; *h*^2^ is the narrow sense heritability and p is the frequency of the favorable genes.
